# Evaluation of Trends in Preexposure Prophylaxis Prescriptions During the First 6 Months of the COVID-19 Pandemic in New York State

**DOI:** 10.1001/jamanetworkopen.2022.4065

**Published:** 2022-03-28

**Authors:** Thomas J. O’Grady, James M. Tesoriero, Yingchao Yuan, Thomas M. Grisham, Seung Jun Seo, Charles J. Gonzalez, Johanne E. Morne

**Affiliations:** 1 AIDS Institute, New York State Department of Health, Albany

## Abstract

This cohort study uses time-series models to estimate preexposure prophylaxis prescription trends during the first 6 months of the COVID-19 pandemic in New York State in 2020.

## Introduction

New York State was disproportionally affected in the early stages of the COVID-19 pandemic, with approximately 462 000 cases and 32 500 deaths by September 30, 2020.^1^ The state has long been an HIV epicenter, and 2019 Centers for Disease Control and Prevention surveillance data indicate that New York State has the highest number of persons living with diagnosed HIV per capita in the US.^2^ There is limited evidence that the early pandemic had a negative association with preexposure prophylaxis (PrEP) access.^3,4^ PrEP is a core pillar in New York State’s effort to end AIDS as an epidemic, making it critical to assess PrEP trends throughout the COVID-19 pandemic. Time-series models were created to estimate the association of the first 6 months of the pandemic with PrEP prescription trends.

## Methods

Deidentified PrEP prescription fill data in New York State from October 20, 2018, to September 27, 2020, were extracted from the Symphony Health IDV data platform by applying a validated PrEP algorithm (eMethods in the Supplement). Extracted data were converted into weekly PrEP prescription fills for the total population and new PrEP initiators. PrEP prescription fill counts were calculated by region, sex, and race and ethnicity. This cohort study was deemed by New York State Department of Health institutional policy to be exempt research, satisfying the protection of human participants exemption criterion per 45 CFR §46.101(b)(4). The study followed the Strengthening the Reporting of Observational Studies in Epidemiology (STROBE) reporting guideline.

Interrupted time-series models based on the Box-Jenkins autoregressive integrated moving average (ARIMA) time-series method^5^ were estimated. The timing of the COVID-19 pandemic and related New York State on Pause efforts constituted the intervention. The ARIMA models were built on the results of the stationarity test and analyses of residuals, outliers, and diagnostic statistics. Separate models for total and new PrEP prescriptions (models 3,1,0 and 2,1,0, respectively) were developed for region (New York City and rest of New York State), sex (men and women), and race and ethnicity (Hispanic, non-Hispanic Black, non-Hispanic White, other, and unknown). Race and ethnicity data were important to include because of known disparities in PrEP uptake among Hispanic and non-Hispanic Black individuals. The COVID-19 association was calculated by comparing observed with projected total and new prescription fills during the 6-month early pandemic period. Data extraction and statistical analyses were conducted using SAS, version 9.4 (SAS Institute Inc).

## Results

The characteristics of PrEP prescriptions from weeks beginning on October 21, 2018, to September 27, 2020, for observed and forecasted weekly totals are provided in the Table and Figure. Sharp decreases in observed PrEP prescriptions corresponded to the beginning of the COVID-19 pandemic in 2020. The observed decrease in total and new PrEP prescription fills decreased below the 95% CIs for the respective projected number of prescription fills for each category and lasted for the entire 6-month early pandemic period.

**Table.** Association of the COVID-19 Pandemic on Total and New Preexposure Prophylaxis Prescriptions From Autoregressive Integrated Moving Average Time-Series Intervention Results^a^

Characteristic	No. of PreEP prescriptions			Change in weekly prescriptions, %^e^	Estimated pandemic-related change in of prescriptions, No.^f^
	Prepandemic mean (95% CI)^b^	Pandemic observed mean (95% CI)^c^	Pandemic forecasted mean^d^		
**All prescriptions**					
Total prescriptions	4351 (4305-4397)	4339 (4300-4379)	5094	–14.8	–23 405
Sex					
Women	210 (200-221)	223 (213-232)	243	–8.2	–620
Men	4140 (4096-4186)	4116 (4078-4154)	4853	–15.2	–22 847
Region of residence					
New York City	3048 (3010-3087)	3004 (2971-3038)	3570	–15.9	–17 546
Rest of New York State	1170 (1144-1196)	1230 (1209-1250)	1420	–13.4	–5890
Race and ethnicity^g^					
Black	442 (425-460)	486 (472-499)	563	–13.7	–2387
Hispanic	508 (490-526)	544 (531-556)	614	–11.4	–2170
White	1942 (1906-1978)	1995 (1967-2023)	2369	–15.8	–11 594
Other^h^	191 (179-204)	199 (190-209)	243	–18.1	–1364
Unknown	1268 (1248-1288)	1116 (1096-1136)	1308	–14.7	–5952
**New prescriptions**					
Total prescriptions	268 (256-280)	206 (192-221)	313	–34.2	–3317
Sex					
Women	34 (29-39)	28 (22-34)	37	–24.3	–279
Men	234 (222-246)	178 (165-192)	278	–36.0	–3100
Region of residence					
New York City	171 (161-181)	135 (124-147)	202	–33.2	–2077
Rest of New York State	84 (76-93)	63 (54-71)	98	–35.7	–1085
Race and ethnicity					
Black	30 (25-35)	25 (20-31)	42	–40.5	–527
Hispanic	31 (26-37)	27 (21-32)	43	–37.2	–496
White	93 (84-102)	70 (61-80)	116	–39.7	–1426
Other^h^	10 (6-14)	8 (4-12)	13	–38.5	–155
Unknown	104 (96-112)	76 (68-85)	98	–22.4	–682

^a^ All models were best fit as autoregressive integrated moving average (3,1,0) and controlled for US holidays including Independence Day, Thanksgiving, and Christmas. Some models controlled for additional outliers.

^b^ Prepandemic means were calculated by dividing the sum of prescriptions for weeks beginning October 21, 2018, to February 23, 2020, by the total number of weeks from that period.

^c^ Observed early pandemic means were calculated by dividing the sum of prescriptions for weeks beginning March 1 to September 27, 2020, by the total number of weeks from that period.

^d^ Forecasted early pandemic means were calculated by dividing the sum of forecasted prescriptions for weeks beginning March 1 to September 27, 2020, by the total number of prescriptions for that period.

^e^ Changes in weekly PrEP prescriptions were calculated as follows: [(Observed pandemic mean − Forecasted pandemic mean)/Forecasted pandemic mean] × 100. Totals within demographic categories may not add to overall totals because of rounding.

^f^ Estimated pandemic-related changes were calculated as follows: (Observed pandemic mean − Forecasted pandemic mean) × 31-week (3-month) period.

^g^ Race and ethnicity were combined into a mutually exclusive variable that highlighted Hispanic, non-Hispanic Black or African American, and non-Hispanic White prescription fills. Because race and ethnicity data were missing for a large percentage of cases, an unknown race and ethnicity category was also tracked.

^h^ Symphony Health IDV category defined as Asian, Native American, or Pacific Islander.

**Figure.**
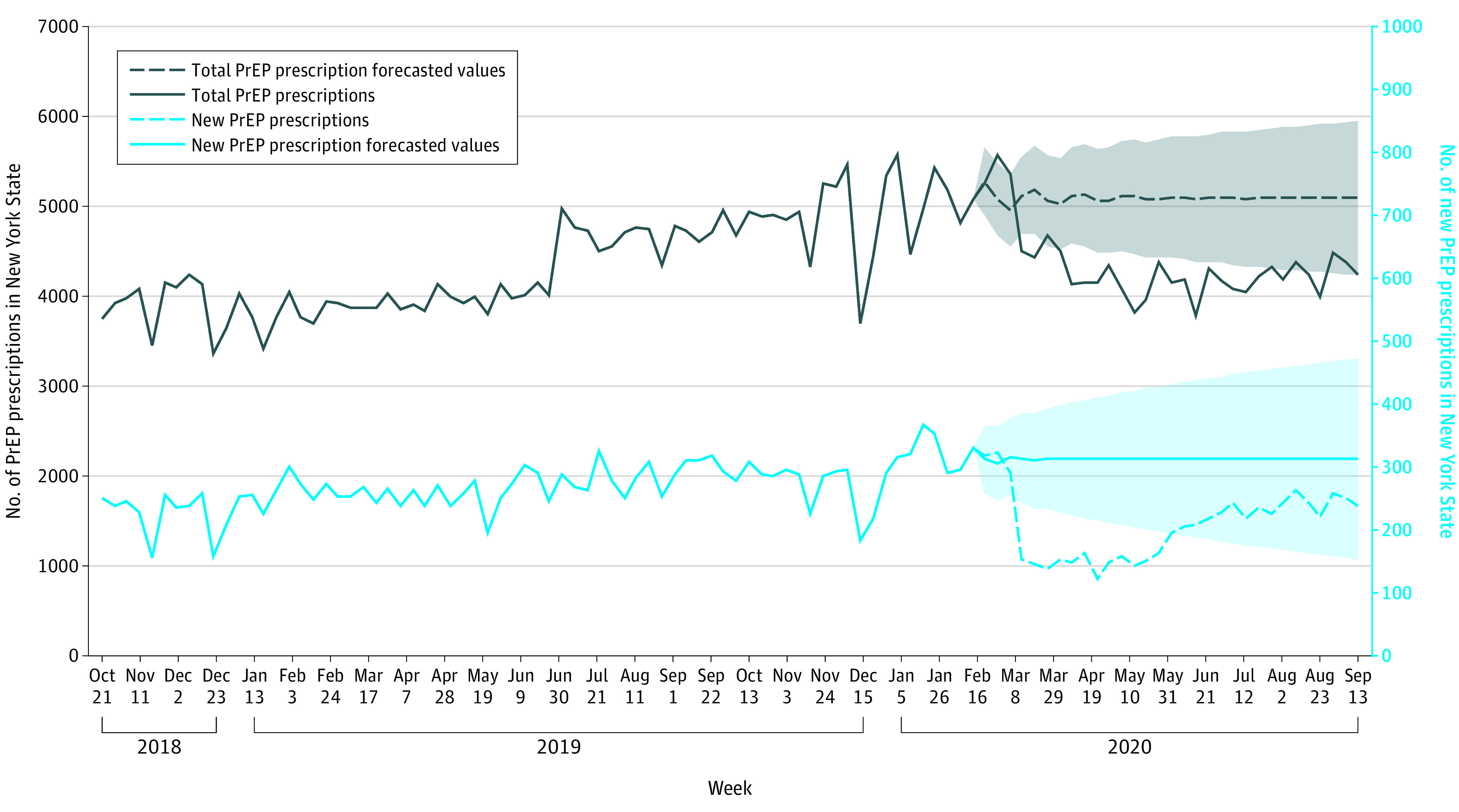
Total and New Preexposure Prophylaxis Prescriptions in New York State for October 21, 2018, to September 27, 2020 Shaded regions represent the 95% CIs for the forecasted total and new preexposure prophylaxis (PrEP) prescriptions during the first 6 months of the COVID-19 pandemic in 2020.

The Table displays the results of ARIMA time-series models estimating total and new PrEP prescription fills by demographic subgroups in New York State. Mean weekly total PrEP prescription fills in New York State decreased by 14.8%, translating to an estimated 23 405 fewer total PrEP prescriptions than projected. Men (−22 847 [−15.2%]) and New York City residents (−17 546 [−15.9%]) experienced the largest decreases. The estimated association of COVID-19 with new PrEP prescription fills was more pronounced than the association with total prescriptions, with new PrEP initiators reduced by a third (−3317 [−34.2%]). This association was observed across all demographic groups.

## Discussion

PrEP use in New York State has increased consistently since the US Food and Drug Administration approved it for HIV prevention in 2012. Continued incNew York State eases in PrEP uptake among men who have sex with men are necessary for New York State to end its AIDS epidemic.^6^ The results of this cohort study suggest an association between the COVID-19 pandemic and PrEP prescription trends in New York State.

Assessing the potential association between sharp decreases in PrEP prescription fills with HIV incidence is complicated. This study’s limitations include a lack of data for a longer period during the COVID-19 pandemic and not knowing how HIV-specific risk behaviors changed among individuals who failed to fill or initiate PrEP prescriptions. Ongoing data review is necessary to understand whether PrEP use trends have stabilized to a “new normal” during the COVID-19 era. More research is needed to understand how longer-term decreased PrEP use will inform New York State policy efforts.

## Notes

Supplement. **eMethods.** Supplemental Methods
eReferences
