# Supplementary material for: Evaluation of Trends in Preexposure Prophylaxis Prescriptions During the First 6 Months of the COVID-19 Pandemic in New York State
Source: JAMA Netw Open. 2022 Mar 28;5(3):e224065. doi: 10.1001/jamanetworkopen.2022.4065 (PMC8961317; doi:10.1001/jamanetworkopen.2022.4065)
Supplement: Supplement. — eMethods. Supplemental Methods eReferences [file jamanetwopen-e224065-s001.pdf]

Supplementary Online Content

O’Grady TJ, Tesoriero JM, Yuan Y, et al. Evaluation of trends in preexposure prophylaxis prescriptions during the first 6 months of the COVID-19 pandemic in New York State. *JAMA Netw Open*. 2022;5(3):e224065. doi:10.1001/jamanetworkopen.2022.4065

eMethods. Supplemental Methods

eReferences

This supplementary material has been provided by the authors to give readers additional information about their work.

© 2022 O’Grady TJ et al. *JAMA Network Open*.

eMethods. Supplemental Methods

Data Source

De-identified PrEP prescription data in NYS from 10/20/2018 to 9/27/2020 were extracted from Symphony Health IDV® by applying a validated PrEP algorithm. This longitudinal data source contains adjudicated prescription, medical, and hospital claims from across the United States for all payment types. Patients were included in the dataset for this analysis based on NYS residence and prescription criteria. The data extracted were then converted into weekly PrEP prescriptions for both the total population and new PrEP initiators by applying a validated PrEP algorithm determine who is prescribed PrEP. The validated PrEP algorithm included cases prescribed emtricitabine and tenofovir disoproxil fumarate (TDF/FTC) or emtricitabine and tenofovir alafenamide (TAF/FTC). The algorithm excluded these prescriptions when they were prescribed as part of HIV treatment, postexposure prophylaxis, or hepatitis B virus treatment.[1]

Statistical Analysis

Interrupted time-series models were created with the timing of the COVID-19 pandemic and related New York on Pause efforts as the intervention. The intervention analysis utilized was a technique developed by Box and Taio based on the Box-Jenkins autoregressive, integrated, moving average (ARIMA) time-series model.[2,3,4] The ARIMA process produces time-series regression models developed for each subpopulation of interest, and accounts for trend and seasonal variation. Models were built on the result of the stationarity test, test and analyses of residuals and outliers, and diagnostic statistics. Separate models for total (ARIMA 3,1,0) and new PrEP prescriptions (ARIMA 2,1,0) were developed for the following demographic

© 2022 O'Grady TJ et al. *JAMA Network Open*.

categories: sex (male, female); race and ethnicity (white non-Hispanic, black non-Hispanic, Hispanic, other, unknown); and region New York City (NYC), Rest of NYS (ROS).

In the ARIMA Time Series Model, Thanksgiving and Christmas weeks were coded as one-week impulse interventions coded as 1 for the weeks of the holidays and 0 for all remaining observations. Large and persistent increases were observed at the beginning of July 2019 and appeared to correspond with the July 4th holiday weekend, so a continuing intervention was coded with a value of 1 for all weeks starting on the week of July 7, 2019 and 0 for all weeks prior. The PrEP Aware Week campaign, a public education campaign, was also modeled as a continuing intervention with a value of 1 from the week of PrEP Aware Week (10/20/2019) to the week leading up to Christmas (12/15/2019) and 0 for all other observations. We compared the observed actual values of total and new PrEP prescriptions in the period from 3/1/2020 to 9/27/2020 with the projected estimates of PrEP prescriptions, had COVID-19 not occurred, for the same time period (3/1/2020 to 9/27/2020) from our models.

Institutional Review Board Statement

This study was deemed by institutional policy to be exempt research, satisfying the Code of Federal Regulations Protection of Human Subjects exemption criterion 45 CFR 46.101(b) Category (4): Research involving the collection or study of existing data, documents, records, pathological specimens, or diagnostic specimens, if these sources are publicly available or if the information is recorded by the investigator in such a manner that subjects cannot be identified, directly or through identifiers linked to the subjects. See https://www.hhs.gov/ohrp/sites/default/files/ohrp/policy/ohrpregulations.pdf

© 2022 O'Grady TJ et al. *JAMA Network Open*.

eReferences

1. Furukawa NW, Smith DK, Gonzalez CJ, et al. Evaluation of Algorithms Used for PrEP Surveillance Using a Reference Population From New York City, July 2016-June 2018. Public Health Rep. 2020;135(2):202-210. doi:10.1177/0033354920904085
2. Box GEP and Taio GC. Intervention Analysis with Applications to Economic and Environmental Problems. Journal of American Statistical Association. 1975; 70:70-79.
3. Box GEP and Jenkins GM. Time Series Analysis: Forecasting and Control. 1976; San Francisco: Holden Day.
4. Soyiri IN, Reidpath DD. Evolving forecasting classifications and applications in health forecasting. Int J Gen Med 2012; 5:381–389.

© 2022 O'Grady TJ et al. *JAMA Network Open*.
